# Supplementary material for: Premature Vascular Aging with Features of Plaque Vulnerability in an Atheroprone Mouse Model of Hutchinson–Gilford Progeria Syndrome with Ldlr Deficiency
Source: Cells. 2020 Oct 8;9(10):2252. doi: 10.3390/cells9102252 (PMC7601818; doi:10.3390/cells9102252)
Supplement: Supplementary file 1 [file cells-09-02252-s001.pdf]

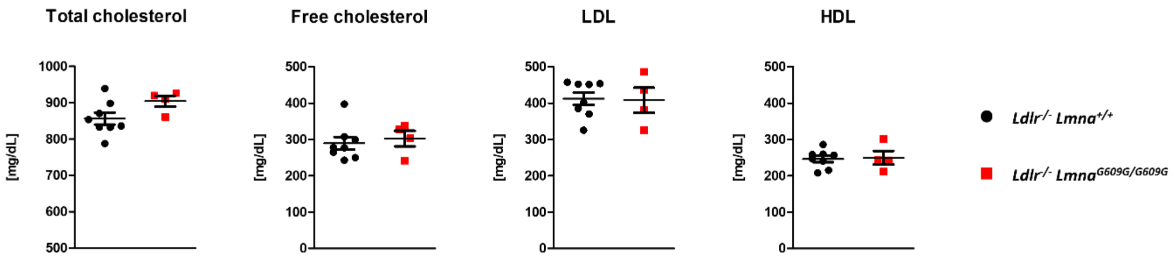

Figure S1. Fat-fed *Ldlr*$^{-/-}$*Lmna*$^{G609G/G609G}$ and *Ldlr*$^{-/-}$*Lmna*$^{+/+}$ mice have similar serum cholesterol levels. Mice were fed a high-fat diet from eight weeks of age and were sacrificed at sixteen weeks of age after overnight fasting. Graphs show serum levels of total cholesterol, free cholesterol, low-density cholesterol (LDL), and high-density cholesterol (HDL) in pooled samples from *Ldlr*$^{-/-}$*Lmna*$^{+/+}$ mice (n = 8) and *Ldlr*$^{-/-}$*Lmna*$^{G609G/G609G}$ mice (n = 4) of both sexes. Each pool was prepared from two-three animals of the same genotype. Data are presented as mean ± SEM. Statistical analysis was performed by two-tailed *t*-test.

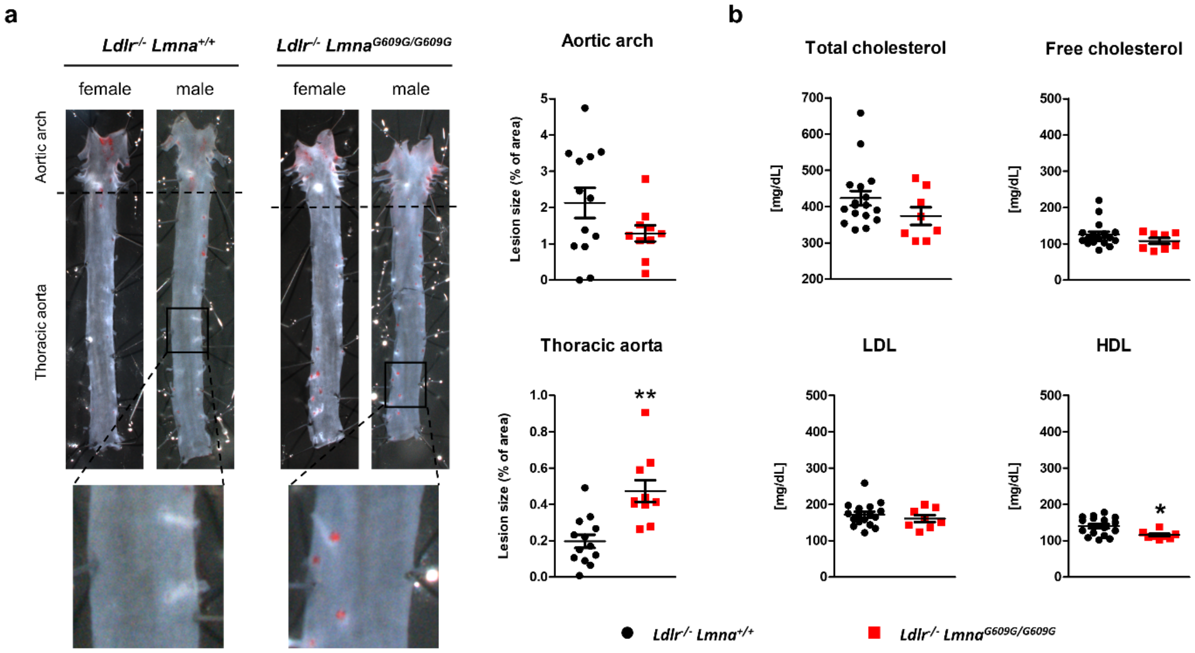

Figure S2. Elevated spontaneous atherosclerosis burden in the thoracic aorta of *Ldlr*$^{-/-}$*Lmna*$^{G609G/G609G}$ mice. Mice were fed normal chow and sacrificed at sixteen weeks of age after overnight fasting. (**a**) Representative images of aortas stained with Oil Red O (ORO) and quantification of atherosclerosis burden (the percentage of the aortic surface positive for ORO) in the aortic arch and thoracic aorta of *Ldlr*$^{-/-}$*Lmna*$^{+/+}$ mice (n = 13) and *Ldlr*$^{-/-}$*Lmna*$^{G609G/G609G}$ mice (n = 10) of both sexes. (**b**) Serum total cholesterol, free cholesterol, low-density cholesterol (LDL), and high-density cholesterol (HDL) in individual and pooled samples (when individual sample volume was insufficient) from *Ldlr*$^{-/-}$*Lmna*$^{+/+}$ mice (n = 17) and *Ldlr*$^{-/-}$*Lmna*$^{G609G/G609G}$ mice (n = 8) of both sexes. Pools were prepared from 2-3 animals of the same genotype. Data are presented as mean ± SEM. Statistical analysis was performed by two-tailed *t*-test with Welch´s correction in (a) and by two-tailed *t*-test in (b). *, p<0.05; **, p<0.01.

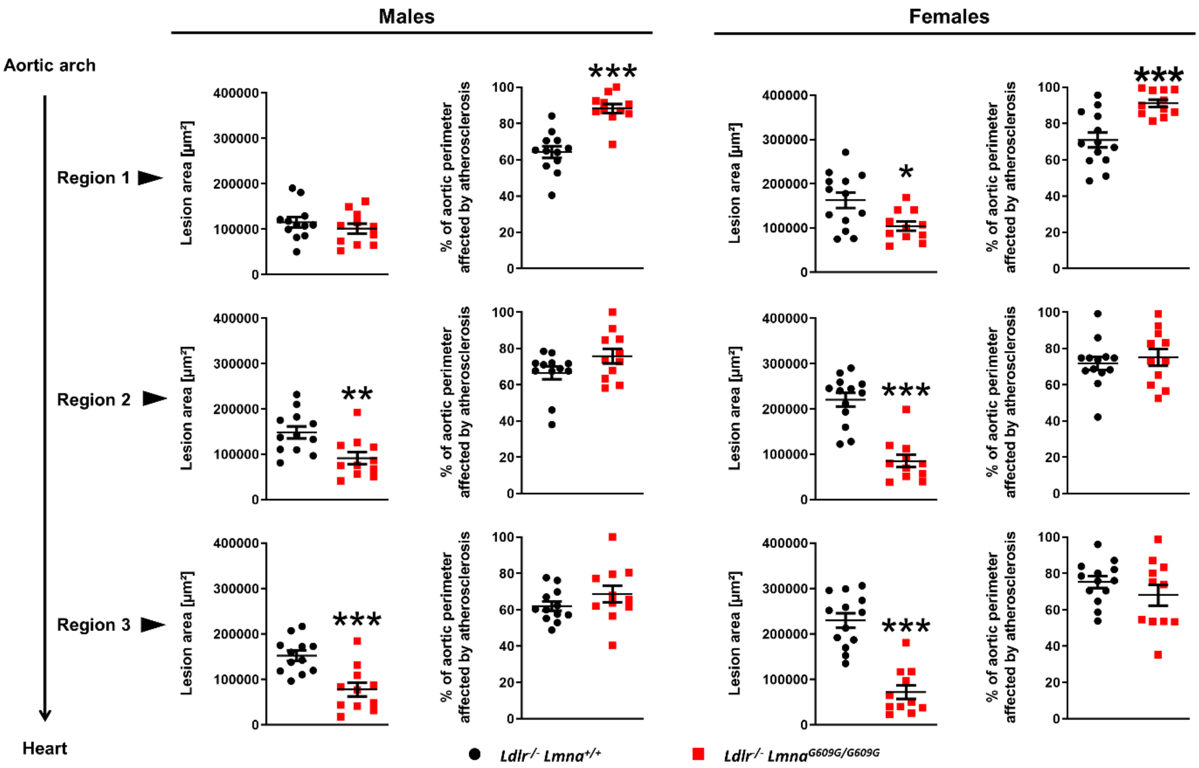

Figure S3. Characterization of atheromas in different aortic root regions of male and female fat-fed *Ldlr-/-Lmna+/+* and *Ldlr-/-LmnaG609G/G609G* mice. Mice were fed a high-fat diet from eight weeks of age and were sacrificed at sixteen weeks of age. The upper part of the heart (containing the aortic root) was sectioned and stained with Masson trichrome. Three aortic root regions were analyzed: at the beginning (Region 1), in the middle (Region 2), and at the end of the aortic valve (Region 3). Graphs show plaque area and the percentage of the aortic perimeter affected by atherosclerosis in *Ldlr-/-Lmna+/+* mice (n = 12 males; n = 13 females) and *Ldlr-/-LmnaG609G/G609G* mice (n = 11 males; n = 13 females). Data are presented as mean ± SEM. Statistical analysis was performed by two-tailed *t*-test. *, $p<0.05$; **, $p<0.01$; ***, $p<0.001$.

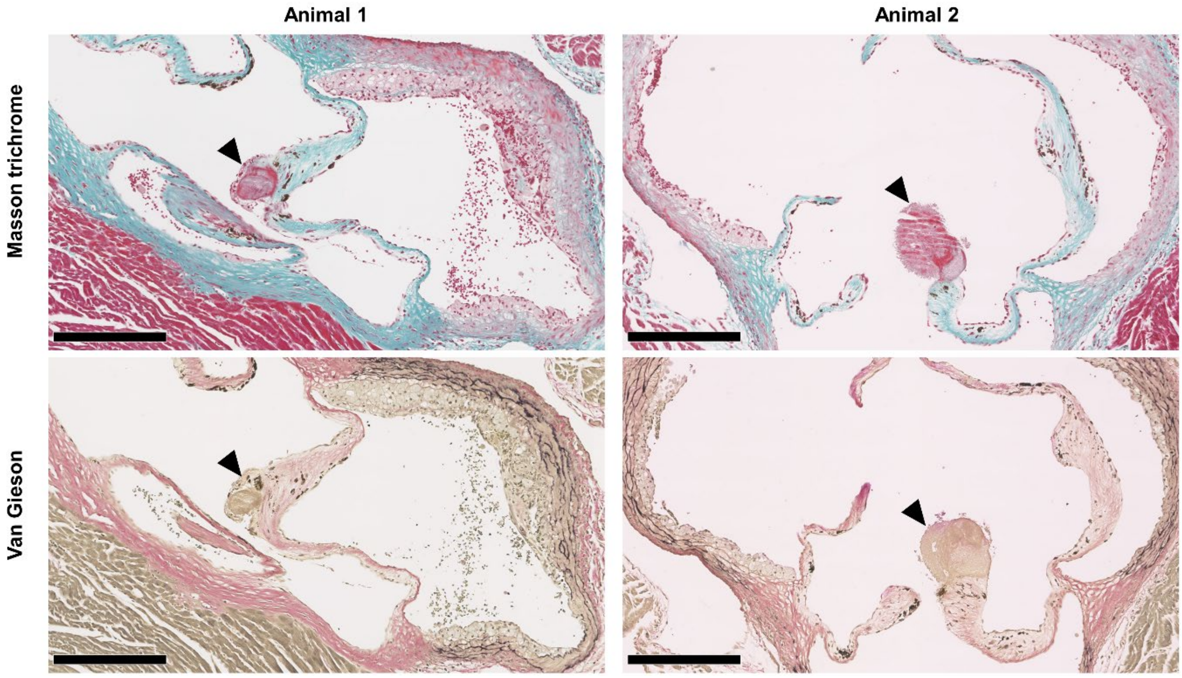

Figure S4. Signs of thrombosis in fat-fed *Ldlr*$^{-/-}$*Lmna*$^{G609G/G609G}$ mice. Mice were fed a high-fat diet from eight weeks of age and were sacrificed at sixteen weeks of age after overnight fasting. The upper part of the heart (containing the aortic root) was sectioned and stained with Masson trichrome and Van Gieson stains. Images show aortic root sections of *Ldlr*$^{-/-}$*Lmna*$^{G609G/G609G}$ mice showing evidence of thrombus formation (arrowhead). Scale bar, 250 µm.

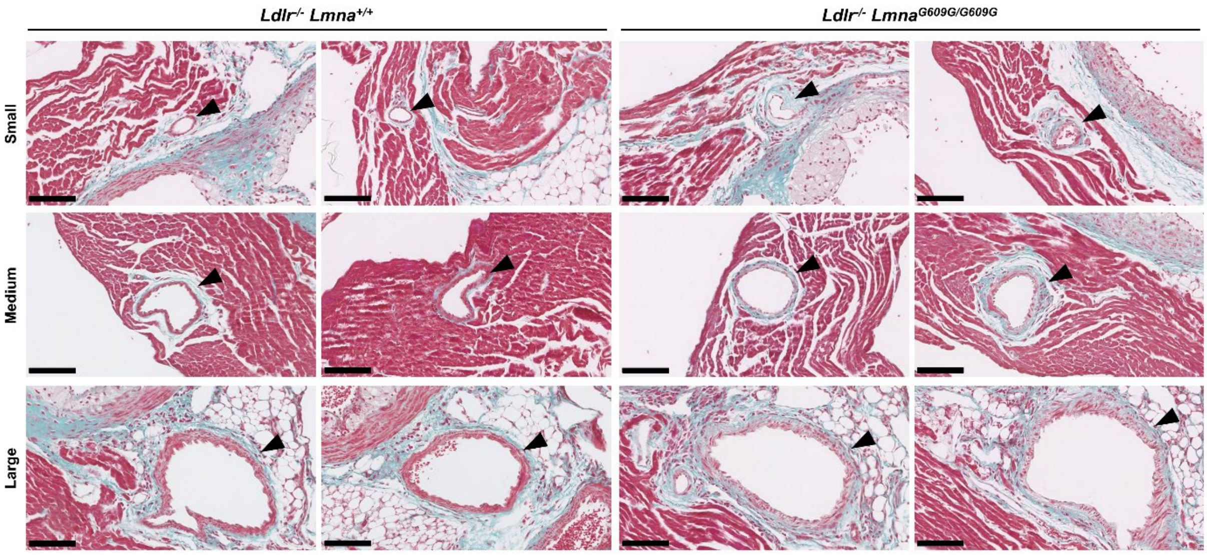

Figure S5. Medial smooth muscle cell loss and adventitial thickening in the coronary arteries in fat-fed *Ldlr*$^{-/-}$*Lmna*$^{G609G/G609G}$ mice. Mice were fed a high-fat diet from eight weeks of age and were sacrificed at sixteen weeks of age after overnight fasting. The upper part of the heart (containing the aortic root) was sectioned and stained with Masson trichrome. Images show representative examples of small, medium, and large coronary arteries (arrowheads) in *Ldlr*$^{-/-}$*Lmna*$^{+/+}$ and *Ldlr*$^{-/-}$*Lmna*$^{G609G/G609G}$ mice. Scale bar, 100 μm.

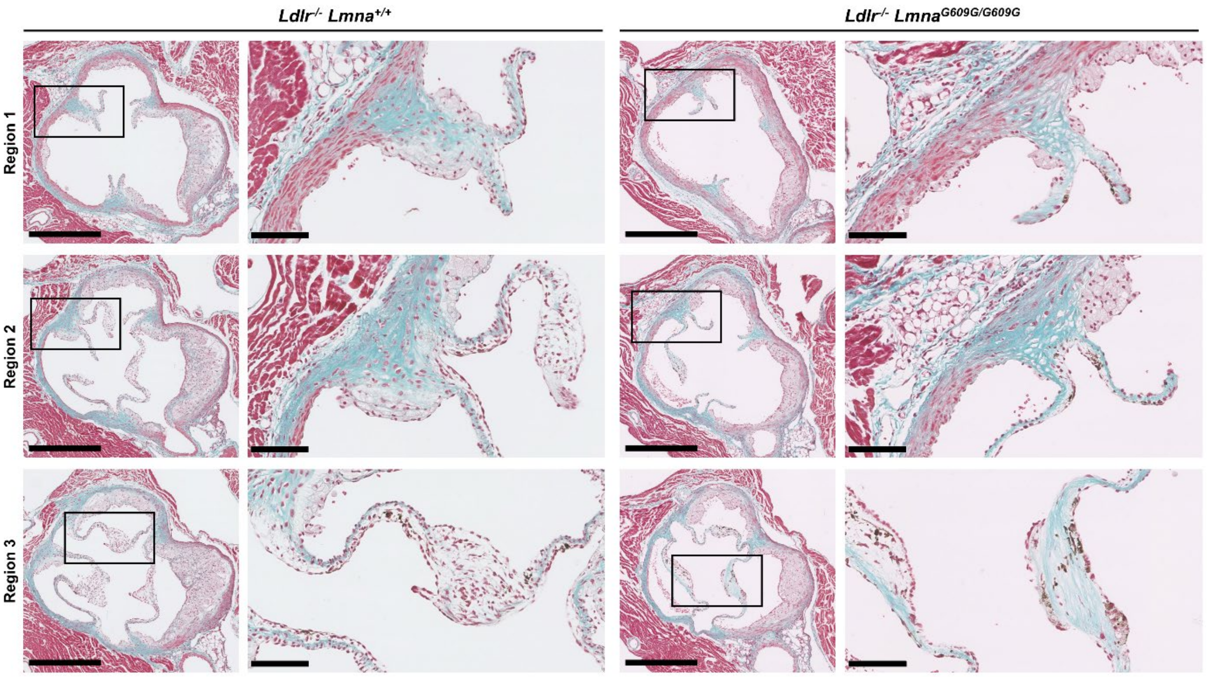

Figure S6. Fibrosis and reduced cellularity in the aortic valve in fat-fed *Ldlr*$^{-/-}$*Lmna*$^{G609G/G609G}$ mice. Mice were fed a high-fat diet from eight weeks of age and were sacrificed at sixteen weeks of age after overnight fasting. The upper part of the heart (containing the aortic root) was sectioned and stained with Masson trichrome. The images show three aortic root regions (at the beginning, middle, and end of the aortic valve) in *Ldlr*$^{-/-}$*Lmna*$^{+/+}$ and *Ldlr*$^{-/-}$*Lmna*$^{G609G/G609G}$ mice. The rectangles in the low magnification images on the left indicate the areas shown at higher magnification on the right. Blue staining color indicates collagen, and brown/dark red spots are nuclei. Scale bars, 500 µm (non-magnified images) and 100 µm (magnified images).

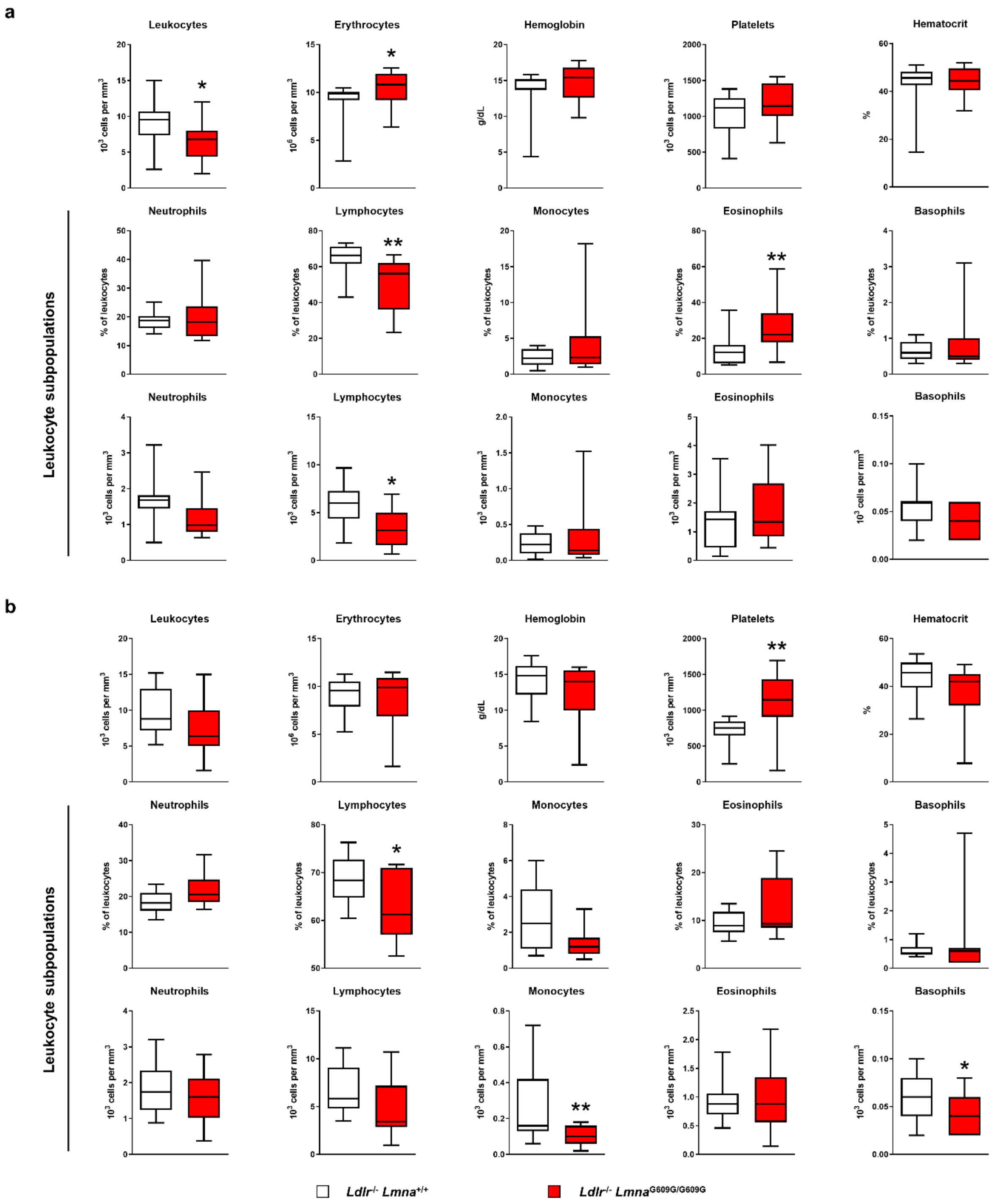

Figure S7. Hematological parameters in fat-fed *Ldlr*$^{-/-}$*Lmna*$^{+/+}$ and *Ldlr*$^{-/-}$*Lmna*$^{G609G/G609G}$ mice. Starting at eight weeks of age, mice were fed high-fat diet for eight weeks. Blood was withdrawn when mice were 16 weeks old after overnight fasting. (**a**) Hematological parameters in male *Ldlr*$^{-/-}$*Lmna*$^{+/+}$ mice (n = 12) and *Ldlr*$^{-/-}$*Lmna*$^{G609G/G609G}$ mice (n = 11). (**b**) Hematological parameters in female *Ldlr*$^{-/-}$*Lmna*$^{+/+}$ mice (n = 13) and *Ldlr*$^{-/-}$*Lmna*$^{G609G/G609G}$ mice (n = 11) mice. Data are presented as median with interquartile range and minimum and maximum values. Statistical analysis was performed by two-tailed Mann-Whitney test. *, $p<0.05$; **, $p<0.01$.
